# Reliable Road Scene Interpretation Based on ITOM with the Integrated Fusion of Vehicle and Lane Tracker in Dense Traffic Situation

**DOI:** 10.3390/s20092457

**Published:** 2020-04-26

**Authors:** Jinhan Jeong, Yook Hyun Yoon, Jahng Hyon Park

**Affiliations:** Department of Automotive Engineering, Hanyang University, Seoul 04763, Korea

**Keywords:** automated driving system (ADS), sensor fusion, multi-lane detection

## Abstract

Lane detection and tracking in a complex road environment is one of the most important research areas in highly automated driving systems. Studies on lane detection cover a variety of difficulties, such as shadowy situations, dimmed lane painting, and obstacles that prohibit lane feature detection. There are several hard cases in which lane candidate features are not easily extracted from image frames captured by a driving vehicle. We have carefully selected typical scenarios in which the extraction of lane candidate features can be easily corrupted by road vehicles and road markers that lead to degradations in the understanding of road scenes, resulting in difficult decision making. We have introduced two main contributions to the interpretation of road scenes in dense traffic environments. First, to obtain robust road scene understanding, we have designed a novel framework combining a lane tracker method integrated with a camera and a radar forward vehicle tracker system, which is especially useful in dense traffic situations. We have introduced an image template occupancy matching method with the integrated vehicle tracker that makes it possible to avoid extracting irrelevant lane features caused by forward target vehicles and road markers. Second, we present a robust multi-lane detection by a tracking algorithm that incudes adjacent lanes as well as ego lanes. We verify a comprehensive experimental evaluation with a real dataset comprised of problematic road scenarios. Experimental result shows that the proposed method is very reliable for multi-lane detection at the presented difficult situations.

## 1. Introduction

The Euro NCAP, NHTSA and ISO have published assessment protocols to meet the proper criteria of the well-known Advance Driver Assistance System (ADAS). The assessment protocols have been renewed almost every year and have become more sophisticated [1]. These documents contain the main fundamental functions of road environment perception to perform ADAS functionalities, like Adaptive Cruise Control, Automatic Emergency Braking and Lane Keeping System. However, each level of the automated driving system (ADS) has an Operational Design Domain (ODD) which means the system is not capable of constant full driving automation since these protocols are subject to the current production line for automakers [2]. To move towards advanced ADS, enormous surges of autonomous driving technologies have focused on the perception of the road environment as well as vehicle control.

Among the autonomous driving technologies, road environment perception requires the ability to use onboard sensors to extract lane markers, road users (like vehicles and pedestrians), and infrastructure (like traffic signs and traffic structures). Especially, the most important but basic research fields are lane and vehicle detection and tracking. We can currently utilize these two states of ego lane and vehicles to adjust the spacing/speed control and the lane keeping system which corresponds to level-2 driving automation out of the 6 levels of driving automation [3]. To advance to level-3 or ultimately full AD, the system requires the ability to overcome issues with reliable and robust multi-lane detection and tracking in complex environments. Especially, the adjacent lanes deliver more specific information to the ADS module to reliably be able to perform accurate and comfortable driving. For example, if the system can predict paths of the surrounding vehicles through the road geometry from lanes, including adjacent lanes, the system can take more safety actions in advance depending on the road scene interpretation. Therefore, to realize highly-advanced ADS technologies, such as planning driving strategies in a complex environment, the perception module must provide key driving information about the accurate lane information, including adjacent lanes, to situational awareness processes.

Most existing lane detection research has concentrated on securing robustness to environmental factors, such as various challenging road types, and shadow and light conditions. Several important studies have explored to multi-lane detection [4,5,6,7,8,9,10,11]. However, there is still a lack of research on adjacent lane detection in the cases that lane features are disturbed by heavy traffic situations or poor visibility. These factors are a critical problem for the previous lane tracker frameworks since most tracking processes rely on the given sequence of image frames. In this paper, we focus on resolving the problems in the extraction of lane candidate features due to occlusion and disturbing features caused by the presence of traffic, road markers, and shadows. We describe the poor extraction of lane candidate features originating from traffic, especially, when the adjacent lane of the road and road markers pose bad influential factors on the process of lane model fitting.

The proposed framework for multi-lane tracking is divided into two main parts: one is an image template occupancy matching (ITOM)-based lane tracker, the other is a frame-level detection approach [6]. Each part was applied with our practical data fusion techniques that combine the vehicle tracker and lane tracker to obtain improvement in managing the adjacent lane as well as the ego lane with an experimental dataset acquired from onboard sensors. Our approach has main contributions of a novel framework of multi-sensor data fusion for a maximum four-lane tracker in the following aspects:

- The state-of-the-art research regarding multi-lane detection focuses on a variety of challenging multi-lane detection scenarios. However, there is a lack of intense research on multi-lane detection and tracking regarding complex road environments for the situational awareness of AD applications. We define three bad influential factors, which are the presence of traffic, road markers, and shadowy road conditions for the four-lane tracker. To overcome this problem, we propose a novel framework for the lane-tracker method integrated with a vehicle tracker consisting of a camera and a radar.
- To assure the robustness of the proposed method to extract lane candidate features, we present two main contributions to the framework for multi-lane tracking. The first is to remove those two key influential factors with the integration of the vehicle tracker and the multi-lane tracker through the ITOM we have introduced. The other is to enhance the performance of the algorithm for extracting lane candidate features in the case of shadowy conditions through a frame level detection approach.
- To assure consistency for the states with multi-lanes, a method for frame-level management of multi-lane detection and tracking is introduced. For multi-lane detection, we design a specified feature extraction function and present a method to select the right feature among the lane candidate features in the current frame. Finally, the states of the multi-lane scenario are framed in a managed condition that it can adjust parameter changes and manage the lane track histories for the multi-lane tracker.

This specialized road scene interpretation method will be discussed throughout the paper. The rest of this paper is organized as follows. Section 2 will explore and summarize related works. Section 3 describes the system overview, including the problem definition, algorithm overview, and problem formulation. The main algorithm is divided into two parts, with the method described in detail in Section 3 and Section 4. The experimental results are presented in Section 5. Finally, the conclusions can be found in Section 6.

## 2. Related Work

Recently, as the ADS techniques have advanced, the need for reliable road scene interpretation, including multi-object detection and tracking methods, has steadily increased. Among these methods, vision-based lane detection and tracking have been stated as one fundamental component of ADS functionalities [2]. In brief, previous research has mainly focused on robust and accurate lane detection. Regarding the specific details in the related works, the lane detection scope can be divided into two parts; single-lane detection and multi-lane detection, as shown in Table 1. According to the detection scope, various approaches for lane detection have been conducted considering road types and conditions with different strategies and characteristics. In single-lane detection, there are various driving situations including from simple road condition [12] to urban areas [13,14], and road environmental difficulties with more complex scenarios [15,16,17,18]. For robustness in lane detection and tracking, parameter estimation based on probabilistic approaches in the form of tracking, using the well-known Kalman Filter (KF) and particle filter, should be conducted. The use of the KF, which is a well-known clothoid lane model-based approach that uses lane features to fit lane models, offers prediction and the smoothing of outliers. However, it is clear that the single model-based approach is not the best choice due to the frequently poor quality of lane features. Therefore, a combination with a multi-model tracking algorithm [12,19] or a feature-based approach, like the ones presented using particle filters [19,20], integrated detection and tracking in the usage of particle filters in [13,16], provides a way to handle the discontinuous road conditions. In [16], Kim introduced a method to find robust lane-boundary hypotheses combining the Random sample consensus (RANSAC) algorithm for detection and a particle filter for tracking. Some other approaches have also proposed a robust lane detection based on the M-estimator Sample Consensus (MSAC) [17], which has a better performance than the RANSAC method for lane model fitting [15]. Recently, several methods based on deep learning for single lane detection have been proposed in [18,21]. Multi-lane detection needs more sophisticated algorithm due to the unknown number of lanes and increase complexities. For a multi-lane feature clustering and association, a method based on conditional random field (CRF) [4,5], lane stability optimization [6], and homgraphy matrix estimation [7,10] are presented. For a multi-lane tracking, particle filter is used considering the dependencies of multiple lane geometric constraints in [8,11], and spline model based KF is also presented [9]. On the other hand, the previous study deal with a robustness on the occlusion and shadow for a short time. For a consideration of complex road environment in practical dense traffic, multi-sensor data fusion to combine data that is not accessible from an individual sensor is inevitable [22,23,24,25]. Over the past few decades, researchers have made substantial progress in multiple research fields studying object tracking. In this paper, we propose a novel framework of multi-sensor data fusion for a multi-lane tracker that works well in a dense road scene interpretation.

## 3. System Overview

### 3.1. Multi-Sensor Data Fusion Framework

**Figure 1:**
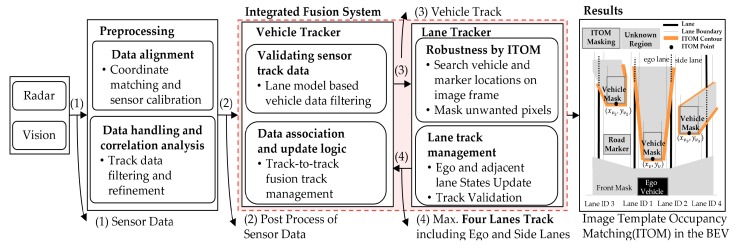
System overview of the multi-sensor data fusion framework.

The main goal of this research is to detect and track a maximum of four-lanes through an integration of road scene information including forward vehicle tracking. As related works have shown in Section 2, it is difficult to handle an algorithm for multi-lane detection that covers all of the various scenarios. Most studies solve the difficulties to combine detection and tracking or apply methods with known robust estimation methods under the tracking process [12,13,14,15,16]. In this paper, the proposed multi-lane tracker framework has key processes for the robust extraction of lane candidate features, including the preprocessing of multi-sensor data, with the integration and fusion of the vehicle tracker and lane tracker. The integrated fusion system with the vehicle tracker compensate for the regions of unwanted feature points that make multi-lane detection difficult in the top-view image frame of the flowchart in Figure 1, which will be explained in detail in Section 4.

● Preprocessing

**Figure 2:**
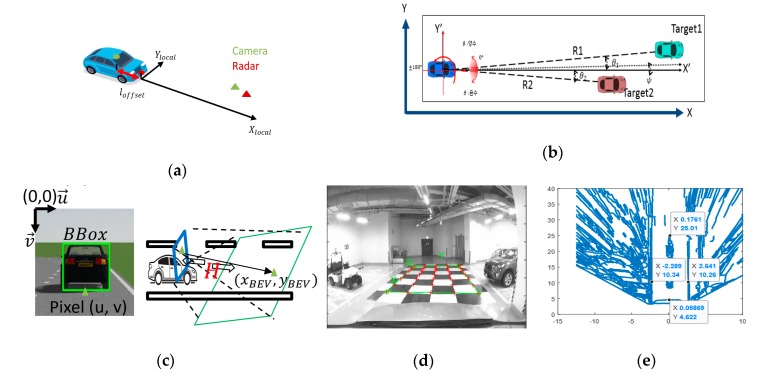
Multi-sensor coordination alignment process: (**a**) multi-sensor coordinate matching, (**b**) radar coordinates, (**c**) transformation of image frame to top-view frame, (**d**) camera calibration, (**e**) transformation into vehicle coordinates.

The coordinate systems are aligned with the point of origin of the front-centered-ground of the ego vehicle which follows a standard SAE coordinate system, but differentiates the center of the rear axis from the front-centered-ground to handle all of the sensor data intuitively in Figure 2a. The *X*-axis is positive toward the front and the *Y*-axis is positive toward the left side on the vehicle. The coordinate matching for each sensor should be conducted since they are installed at different mounting positions. For a radar sensor in Figure 2b, it is easy to employ the coordinate by a simple transition from polar coordinates to vehicle coordinates. However, for the camera in Figure 2c, the image frame should be rectified into the top-view image, which can be aligned with the same vehicle coordinates by Inverse Perspective Mapping (IPM) [15,16,17]. Through the image transformation, we intuitively interpret a road scene situation with the integrated vehicles and lanes information at the same time. To obtain a top-view image or bird-eye view (BEV), we need to find a homography matrix by the camera calibration process in Figure 2d. For clarity, each sensor transformation is constructed as follows:

- The transformation from each sensor frame to the vehicle local frame:
  (1) $$vehicle\;local\;frame:\;\left\{X_{local},Y_{local}\right\},$$
- Typical installation positions of the camera and radar sensor:
  (2) $$\mathrm{Radar}:\;\left[\begin{array}{c} X_{r\_local}\\ Y_{r\_local} \end{array}\right]=\left[\begin{array}{c} R_{r}cos\left(\theta_{r}\right)\\ R_{r}sin\left(\theta_{r}\right) \end{array}\right],$$
  (3) $$\mathrm{Camera}:\left[\begin{array}{c} X_{v\_local}\\ Y_{v\_local} \end{array}\right]=\left[\begin{array}{c} x_{BEV}+l_{offset}\\ y_{BEV} \end{array}\right],$$
- Image transformation
  (4) $$A\;\mathrm{camera}\;\mathrm{matrix}:\;H_{3\times 4}=\left[\begin{array}{ccc} fx & 0 & cx\\ 0 & fy & cy\\ 0 & 0 & 1 \end{array}\right]{\left[R|T\right]}_{3x4},\;$$
  (5) $$A\;\mathrm{homography}\;\mathrm{transformation}\;\mathrm{matrix}:\;Trans=H_{3\times 4}\left(:,\left[1\;2\;4\right]\right),$$
  The camera intrinsic parameters (fx,fy, cx, and cy) and extrinsic parameters (R: rotation, T: Translation matrix) are obtained from the process of the camera calibration in Figure 2d.

Once we obtain the homography matrix through the Equations (4) and (5), we can find a point (u,v) in the image frame corresponding to the point $\left(x_{BEV},y_{BEV}\right)$ which indicates Figure 2c,e as follows:

Vehicle to image transformation:

(6) $${\left(u^{\prime },v^{\prime },\alpha \right)}^{T}=Trans*{\left(x_{BEV},y_{BEV},1\right)}^{T}\;{\left(u,v\right)}^{T}={\left(u^{\prime }/\alpha ,v^{\prime }/\alpha \right)}^{T},$$

Image to vehicle transformation:

(7) $${\left(x^{\prime },y^{\prime },\alpha \right)}^{T}=Trans^{-1}*{\left(u,v,1\right)}^{T}\;{\left(x_{BEV},y_{BEV}\right)}^{T}={\left(x^{\prime }/\alpha ,y^{\prime }/\alpha ,1\right)}^{T},$$

### 3.2. Experimental Setup

**Figure 3:**
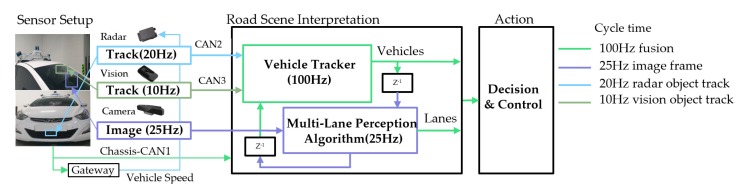
The multi-rate and multi-sensor fusion flow chart.

The test vehicle is equipped with a radar, vision module, and a camera. The self-collected datasets are acquired for evaluating the proposed multi-lane tracker in a variety of complex road conditions and the validation process was conducted in post-process. The onboard perception module obtains the radar object tracks, vision lane/object tracks, and ego vehicle data from the CAN bus and the current image frame at different cycle times. As shown in Figure 3, the multi-rate and multi-sensor fusion flow chart depicts the test sensor setup and how the system works with a different sensor set.

- A 76-77GHz (174 m, +/−10°) and mid-range (60 m, +/−45°) delphi-ESR1.1 radar provides simultaneous multimode electronic scanning simultaneously. However, we are only concerned with the object’s range $\left(R_{r}\right)$, azimuth $\left(\theta_{r}\right)$, and speed $\left(v_{r}\right)$ data up to 64 targets within the region of interest of the extent of the image frame (35 m).
- A Mobileye 630 vision module detects and tracks four-lane and vehicle positions up to 64 target tracks with a horizontal FOV of $47^{\circ }$. We take object position track $\left(x_{V},y_{v}\right)$ for the sake of facile fusion with the radar track instead of vehicle detection from the image frame. Furthermore, we can easily compare four-lane tracks with our integrated lane tracker method.
- A RealSense SR-300 camera offers 1920 × 1080 pixels with a horizontal FOV of 73°, with which we interpret and understand the road scene. We have downsized the image by 640 × 480 pixels for improving computational efficiency with performing appropriate performance for the situational awareness. The image frames are mainly used for multi-lane detection and integration with the vehicle tracker. The feature points in the vehicle coordinates are derived from the rectified top-view image frame as described in Section 3.1.

## 4. Integrated Fusion of the Vehicle and Lane Tracker

**Figure 4:**
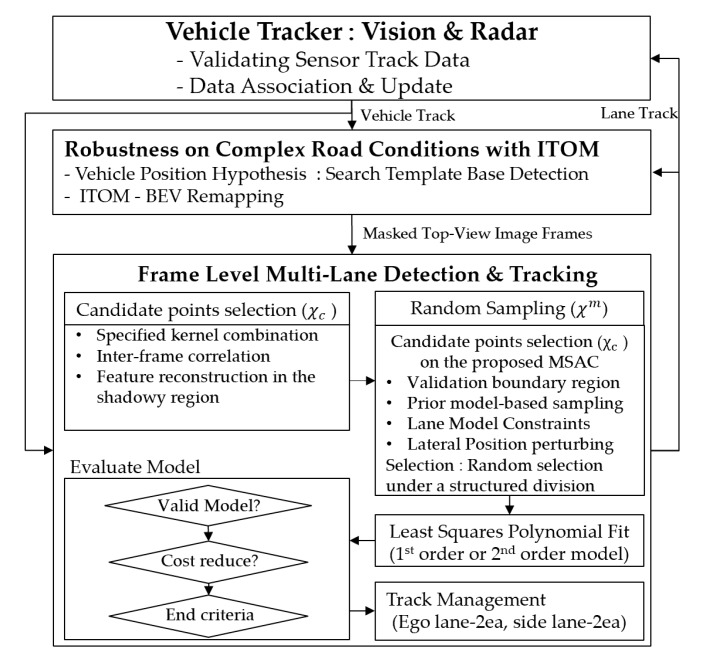
The robust multi-lane detection and tracking algorithm.

In the case of a presence of vehicles to the front or side, or road markers and shadows, it is impossible to consistently track multiple lanes consistently without any complementary solutions. We present an integrated fusion method for the multi-lane tracker and vehicle tracker that are complementary to the process of updating each track state. The robust multi-lane detection and tracking algorithm consists of four main steps: a vehicle tracker with vision and radar, robustness under complex road conditions with ITOM, frame level multi-lane detection, and multi-lane track management as shown in Figure 4.

### 4.1. Vehicle Tracker

**Figure 5:**
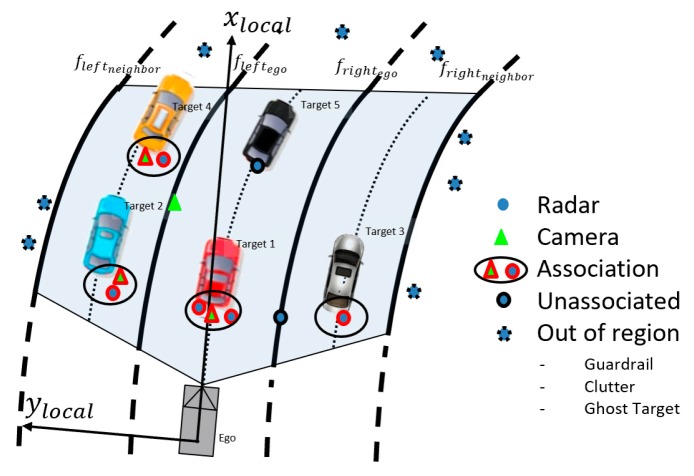
Multi-object data association within region of interest.

In this research, the vehicle tracker is responsible for combining the multi-rate multi-sensor track data fusion. The issues in the vehicle tracker process can be categorized into three steps: validating the track data, data association, and lowered delay of track fusion, which is part of the track-to-track fusion with the radar and vision system [24]. In other words, these datasets have already tracked the object states. One major characteristic is that the radar track includes clutter and noisy radar data, from guardrails, bridges, tunnels, etc., besides road users within the radar sensor range while the vision track only indicates certain vehicles in front of the ego vehicle. Therefore, we should consider the two issues of validating the track data and data association. First, a region of interest (ROI) is set up for validating the track data. The ROI is based on a lane boundary, which is determined by a lane tracker, as shown in Figure 5. In the case of Figure 5, where the lane tracker has four active lanes, the region is restricted to along the adjacent lane boundaries for the lateral direction and a distance limitation for longitudinal direction. The plot describes that the irrelevant radar data, such as guardrails and clutter, can be filtered out with the ROI. Secondly, the track data from each sensor decides whether fused track state and the new incoming track data is admissible inside gating using the Mahalanobis distance which is derived from $\chi^{2}$-distribution [25]. Finally, the track update process follows the work well accomplished by the use of the Kalman Filter [26]. In the track update process, there are three states which are track creation, standby, and confirmed. We take the confirmed track when the vision track comes at the first time of track update states, while we take the standby track when the radar track arrives first due to the uncertainty. That is, the track update rules give priority to the vision track for lowered delay time while updating vehicle track states.

### 4.2. Lane Tracker

**Figure 6:**
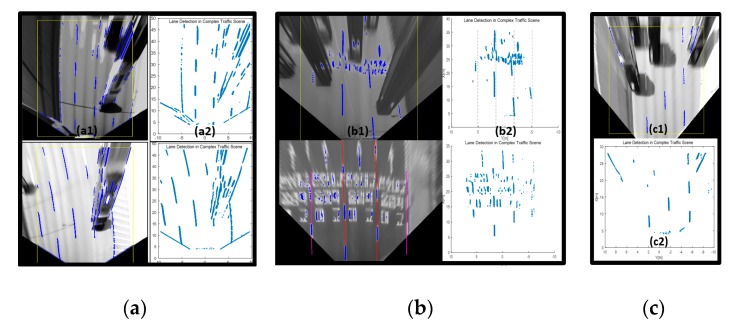
Three bad influential factors for lane detection: (**a**) a challenging scenario for road adjacent lane detection due to a vehicle in the side lane, (**b**) a challenging scenario for road lane detection due to road markers, (**c**) a shadowy road condition, with (a1,b1,c1) lane candidate points from a bird’s-eye view and (a2, b2, c2) lane candidate points in the vehicle coordinates.

As mentioned in the related works, many unfortunate influential factors make lane detection surprisingly difficult, even if the extraction of lane candidate features from a road may seem straightforward. Since most tracking processes depend on the lane candidate features, the previous lane tracker frameworks are easily destroyed by consistently wrong or absent lane candidate features that occur from occlusion, poor visibility, or shadows in dense traffic conditions as shown in Figure 6. While these road conditions can be handled by previous research methods for a short time. ADS must catch adjacent lanes to be able to aware of the current traffic situation in the case of complex road conditions. To accomplish this special case, we present a robust lane tracker that secures adjacent lanes as well as ego lanes while integrating the vehicle tracker. In brief, the proposed lane tracker consists of two main parts: frame-level multi-lane detection and tracking, and securing robustness in complex road condition.

#### 4.2.1. Frame Level Multi-Lane Detection and Tracking

● Lane Candidate Feature Extraction

**Figure 7:**
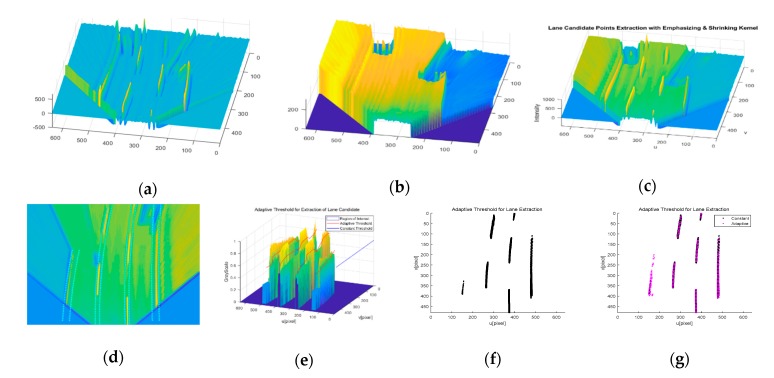
A robust lane feature extraction method, (**a**) emphasizing lane features (indicated by brightness), (**b**) shrinking the road region (darkness), (**c**) combining the two kernels, (**d**) frame level multi-lane region of interest (ROI), (**e**) reconstruction of the shadowy region features with a lane-level adaptive threshold, (**f**) lane feature extraction results without an adaptive threshold, (**g**) lane feature extraction results with an adaptive threshold.

This method requires initial lane candidate feature extraction. The features are extracted using a designed kernel edge filter based on the dark-light-dark (DLD) intensity transition characteristic [14]. For the kernel design, we first transform the image frame to a bird’s-eye view image with IPM. From the rectified image frame, we analyze the lane width in the bird’s-eye view image and design the specified kernel function, which consists of emphasizing the lane feature function ($f_{E}$) and shrinking the road region feature function $f_{S}$. Finally, we can extract the lane candidate features from the specified kernel combination function $f_{c}$, as shown in Figure 7a–c and Equations (8)–(10).

(8) $$f_{E}=conv\left(I,\left[-1\;0_{1\times n}\;2\;\;0_{1\times n}-1\right]\right),$$

(9) $$f_{S}=conv\left(I,\left[1\;0_{1\times n}\;0\;\;0_{1\times n}-1\right]\right)-conv\left(I,\left[1\;0_{1\times n}\;0\;0_{1\times n}\;1\right]\right),$$

(10) $$f_{c}=\alpha f_{E}+f_{S}$$

where conv(I,kernel) means a convolution with image frame I (a 640 × 480 pixel image) and kernel. The α scale factor indicates the amplification of the brightness. However, this extraction method is very unstable under shadows because of the gradient changes over different road environments. To handle the shadow problem, an adaptive threshold has been employed in this work. The process of reconstruction in the shadow region is conducted as shown in Figure 7d–g. Figure 7d represents the multi-lane ROI derived with the information from the front vehicle states and previous lane track states. Within the multi-lane ROI, we take an adaptive threshold for each lane to reconstruct lane candidate features as shown in Figure 7e. Finally, we can obtain well-resolved features of the current frame and we can compare the results in Figure 7f,g.

● Robust Lane Detection and Tracking

The lane model described in [27] is a third order polynomial, clothoid model. In this work, we reduce the order of model to a parabolic or linear model according to feature quality because these models are more stable in complex traffics, and the model can be formularized as follows;

(11) $$\mathrm{Clothoidal}\;\mathrm{model}:\;f_{index}^{3nd}\left(l\right)=y_{0}+\psi l+\frac{C_{0}}{2}l^{2}+\frac{C_{1}}{6}l^{3}$$

(12) $$Parabolic\;modle:\;f_{index}^{2nd}\left(l\right)=y_{0}+\psi l+\frac{C_{0}}{2}l^{2}$$

(13) $$\mathrm{Linear}\;\mathrm{model}:\;f_{index}^{1st}\left(l\right)=\psi l+y_{0}$$

where index indicates the$\;left_{ego}$, $right_{ego}$, $left_{adjacent}$, $right_{adjacent}$ lanes, respectively, and the lane parameters consist of $y_{0}$, ψ, $C_{0}$,${\;C}_{1}$ which are the lateral offset, lane heading, lane curvature, curvature rate, and l is a longitudinal distance.

**Figure 8:**
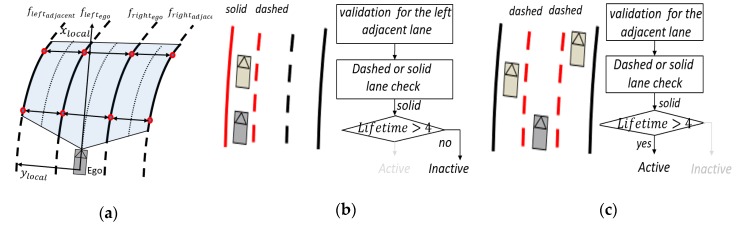
Lane track management, (**a**) subject multi-lane ($left_{ego}$, $right_{ego}$, $left_{adjacent}$, $right_{adjacent}$), (**b**,**c**) two cases of adjacent left lane with lane validation logic.

For multi-lane detection, the proposed approach has been designed to handle more sophisticated individual lane detection based on the MSAC [28,29] under strictly controlled feature selection methods using the frame-level track coherence. Taking advantage of this characteristic, we applied an individual lane feature selection strategy to find the feature cluster more quickly and efficiently as shown in Figure 4. The individual four lanes have their own constraints based on a validation boundary region of the lane model parameters in Equation (12). Since the lane features cannot be changed abruptly, we take advantage of the validation boundary regions defined by the estimated lane parameters in the previous frame where the candidate points are located in the current frame. Through multi-lane track management, we group the subject multi-lane parameters ($left_{ego}$, $right_{ego}$, $left_{adjacent}$, $right_{adjacent}$) and manage them separately, as shown in Figure 8a. Every lane tracker cycle time increment, we check for adjacent lane validations, as shown in Figure 8b,c. We make sure of two check points (lane types and *Lifetime*) for clarity to validate the adjacent lane: a check on whether each ego lane is a dashed or solid lane, and the life time, which represents the number of track update after track creation, of the adjacent lane (*Lifetime* > 4). The reason for finding lane types is to determine whether the ego vehicle is in a driving edge lane, which means the lane no longer exists over the lane boundary. We also double-check any vehicle that has the same direction and velocity with the ego vehicle for third check.

#### 4.2.2. Robustness under Complex Road Conditions

**Figure 9:**
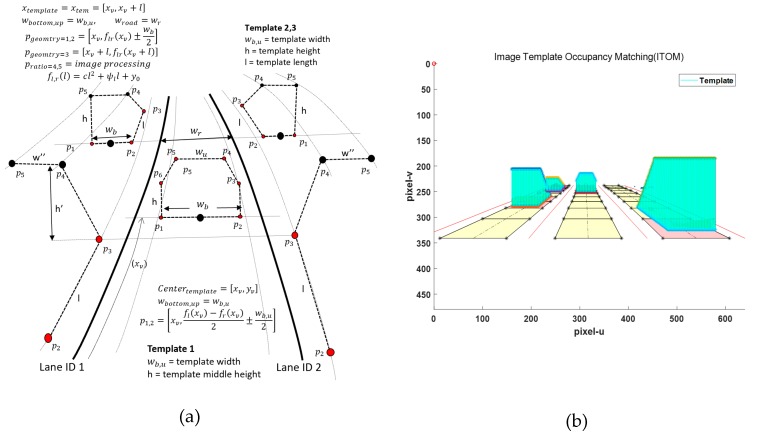
Image template occupancy matching (ITOM) concept plot, (**a**) template design, (**b**) search base template and match on image frame.

**Figure 10:**
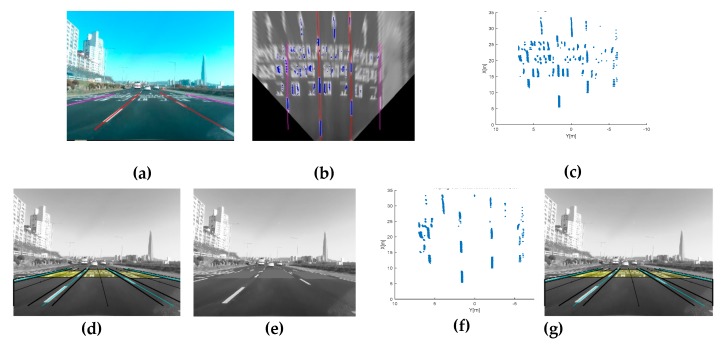
Complex road conditions: ITOM-based road markers elimination. (**a**) Previous multi-lane states of the image frame, (**b**) top-view frame, (**c**) origin features, (**d**) ITOM process, (**e**) remove road marker, (**f**) features in the vehicle coordinates, (**g**) multi-lane detection.

**Figure 11:**
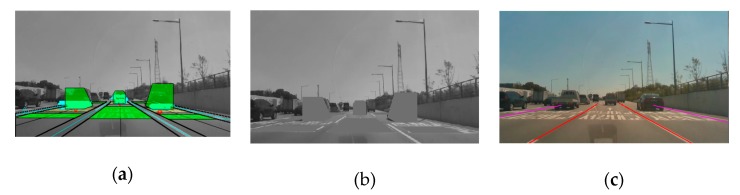
Complex road conditions: (**a**) template-based detection, (**b**) ITOM remapping on the origin image frame, (**c**) robustness in complex road conditions.

In feature-based methods, Otsu thresholding segmentation has often been used and proven in preprocessing [23]. This thresholding method is also useless when the whole image frame is full of bright objects, when most of the image frame region is occupied by road markers, or in the presence of bright cars on the road. Therefore, we intend to mask all these factors to extract brightness intensities only corresponding to the lane features. In order to eliminate these factors, we introduce a novel concept of image template occupancy matching (ITOM). This concept finds a literally occupied region that matches with vehicles or road markers using predefined templates, as shown in Figure 9a. These templates consist of base templates and vehicle templates based on each lane geometry. The base template is responsible for searching hypotheses for road markers or vehicles, as shown in Figure 9b. After finding a hypothesis location, the occupancy-matching procedure is conducted. Each base template determines the occupancy of the vehicle position on the image from the front vehicle states from the vehicle tracker. If there are no matching results with a vehicle, it regards the candidate as a road marker, as shown in Figure 1 (results). The ITOM-based multi-lane tracker results are shown in Figure 10 and Figure 11. In Figure 10a depicts the results of multi-lane detection on an image frame, Figure 10b displays the lane candidate features, Figure 10c depicts noisy lane candidate features in the vehicle coordinates, Figure 10d shows base templates occupied with road markers and the lane geometry, Figure 10e shows the elimination of road makers for each lanes, Figure 10f shows the lane candidate feature with removed road markers, and Figure 10g shows the multi-lane detection results for current frame. In Figure 11, when vehicles and road markers appear at the same time, the ITOM-based multi-lane tracker has successfully eliminated the disturbance factors and parameterized the driving environment.

## 5. Experimental Results

### 5.1. Experimental Methodology

The performance of the reliable road scene interpretation based on ITOM with the integrated fusion of the vehicle and lane tracker in a dense traffic situation is evaluated through mainly self-collected dataset from real driving data in dense traffic conditions. The purpose of this experiment mainly focuses on the specific driving conditions which fall under the consistent traffic occlusion from forward vehicles and the existence of road markers and shadows that yield incorrect lane parameter estimations. The video with a resolution of 640 × 480 from the evaluation dataset are manually labeled using the Matlab ground truth labeler. If the differences between the tracked multi-lanes and the ground truth are within a pre-defined threshold, a true positive detection is counted, otherwise a false negative [9]. The performance metric is as follows: A true positive (TPR) = (the number of detected lanes)/(the number of target lanes), a false positive rate(FPR) = (the number of false positives)/(the number of target lanes) [5,7].

### 5.2. Experimental Results

**Figure 12:**
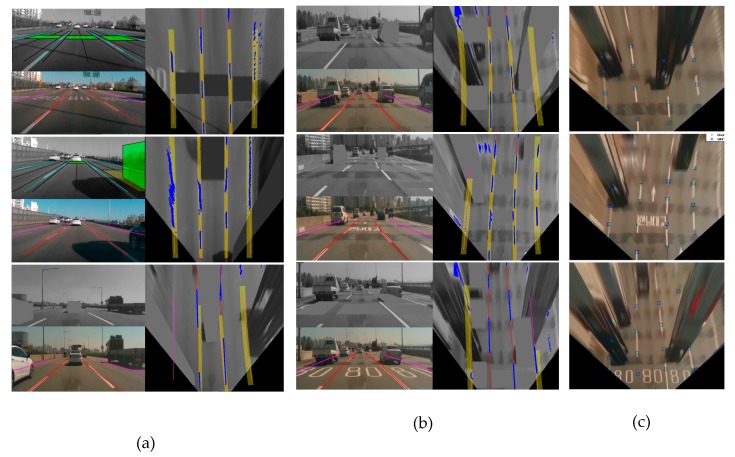
Experimental results and validation (**a**) scenario I: successful masking of the road marker, side vehicle emergence, front, and side vehicles. (**b**) Scenario II: successful masking of a complex scenario, (**c**) ground truth and results from the top view for validation.

We test and verify the multi-lane tracker performance on the normal PC, Intel(R) Core i9-9900KF CPU @ 3.60GHz and 32.00GB RAM. The processing speed is around 25 frame per second (fps). It is a little slow, but it has enough possibility to optimize the proposed algorithm which is reasonable speed for ADs in a real time processor. A total of 3350 frames were tested and the target four-lanes are considered within 35m. The performance metric shows that the overall TPR of the algorithm for identifying lanes, including adjacent lanes, is 99.5 percent and the adjacent lane TPR is 96.8 percent, as shown in Table 2. This is a significant improvement compared to the Mobileye 630 lane tracker results, which has a TPR of only 90 percent and 88.6 percent respectively. We show a variety of the experimental results and validation in Figure 12. The figure describes presence of road marker and multiple vehicle ahead of ego vehicle. We picked specific difficult scenarios in Figure 12 which depict a road marking scenario in first row of (a), vehicle appearance on right or left lane in the rest rows of (a), mixed scenarios of complex road marking and vehicle ahead of ego vehicle in (b), and finally validation with the tracked multi-lanes and ground truth in (c). It is shown that the proposed multi-lane tracker outperformed in the ego lanes and adjacent lanes through disturbing features elimination.

## 6. Conclusions

This work proposed and evaluated a road scene interpretation algorithm based on ITOM with the integrated fusion of the vehicle and lane tracker in a dense traffic situation. The system mainly focused on resolving the problems in the extraction of lane candidate features due to three bad influential factors in complex road conditions under the process of a multi-lane tracker. To overcome this problem, the framework for a multi-lane tracker presents two key processes for the robust extraction of lane candidate features. The first process introduces an elimination process of bad influential factors including the presence of vehicles and road markers in the current top-view image frame through the proposed ITOM method. In this process, we consider validating track data, data association, and the radar track-to-vision track fusion method which gives priority to vision track for a lower delay time while updating vehicle track states. In the second process, the proposed robust methods for frame-level multi-lane detection and tracking are presented, including the extraction of lane candidate features under shadows, a frame-level lane hypothesis region, and managing the multi-lane tracker using the MSAC algorithm. In the experiment, we have carefully selected dataset to verify specific bothersome scenarios in which the study is interested. The test results showed that the proposed method can significantly improve the detection rates when it comes to the adjacent lanes under complex road conditions. Through this framework, we ensure multi-lane detection and tracking that make further driving strategies with situational awareness in ADs. However, this work needs to check the reliability in much more road scenarios and a variety of road types as well since this work mainly focus on the methodology of robust lane feature extraction and managing in the presence of road marking, vehicles on the road and shadows.

## Figures and Tables

**Table 1:** Various approaches for lane detection.

Research Target Detection Scope	Road Types and Conditions	Strategies and Characteristics
Single-Lane [12,13,14,15,16,17,18,19,20,21]	Challenge scenarios [13,14,15,16,20]	Particle filter [14,19,20], Kalman [12,16], Robust strategies [15,16,17], Deep learning [18,21]
Multi-Lane [4,5,6,7,8,9,10,11]	Challenge scenarios [4,5,6] Adjacent lane [7,8,9,10]	CRF association [4,5], Robust strategies [6,7,10], Particle filter [8,11], Spline EKF [9]

**Table 2:** Various approaches for lane detection.

Measure	TPR			FPR
Type	Ego Lane	Adjacent	Total	Total
ITOM Lane Tracker	0.995	0.968	0.98	0.019
Mobileye 630	0.914	0.886	0.9	0.1
